# Primary Lymphangioma of the Tonsil: A Case Report

**DOI:** 10.1155/2011/183182

**Published:** 2011-05-31

**Authors:** Dimitrios G. Balatsouras, Alexandros Fassolis, George Koukoutsis, Panayotis Ganelis, Antonis Kaberos

**Affiliations:** ENT Department, Tzaneio General Hospital of Pireaus, Afentouli 1 and Zanni, 18536 Piraeus, Greece

## Abstract

Benign tumors of the tonsils occur infrequently. Lymphangiomas are rare congenital tumors of the lymphatic system, and tonsillar lymphangioma is an extremely rare occurrence. Its pathogenesis is uncertain, but history, clinical examination, and histological examination should establish the diagnosis. We present a 17-year-old white male with lymphangioma of the right tonsil. The tonsils were excised and biopsy confirmed the diagnosis. Tonsillar lymphangioma is a rare clinical entity, which should be known to the otolaryngologist, in order to diagnose and treat it appropriately and avoid confusion with tonsillar malignancies.

## 1. Introduction

Benign tumors of the tonsils occur infrequently. The most frequently reported benign tumors of the tonsils are papillomas, angiomas, fibromas, myxomas, lipomas, chondromas, inclusion cysts, and teratogenous cysts [1]. Histological confirmation is essential for diagnosis.

Lymphangiomas are rare congenital tumors of the lymphatic system. Although they are usually present at or around the time of birth, they usually manifest within the first two decades of life. Three types of lymphangiomas in the head and neck region may be distinguished [2]: (1) lymphangioma simplex, which is composed of thin-walled capillary-sized lymphatic channels; (2) cavernous lymphangioma, which in almost half of the cases occurs in the tongue; (3) cystic hygroma.

We report a case of lymphangioma of the tonsil in an otherwise healthy young male, and we briefly review the existing literature.

## 2. Case Presentation

A white 17-year-old male student was admitted to the ear nose throat department following referral from the accident and emergency department of our hospital. The patient presented with a 3-day history of dysphagia that was not associated with pain. He reported greater difficulty in swallowing solids than liquids. Furthermore, during the past weeks his sleep was disturbed due to a sensation of foreign body in the throat, causing him a nonproductive cough. This progressed to difficulty in swallowing his saliva on the day of presentation and prompting him to attend the accident and emergency department. 

Physical examination showed a well-developed white male in good general health with no past medical history or allergies. Blood pressure was 145/77, pulse was 78, SaO_2_ was 98, respiratory rate was 16/min, and temperature was 36.4°C. 

**Figure 1:**
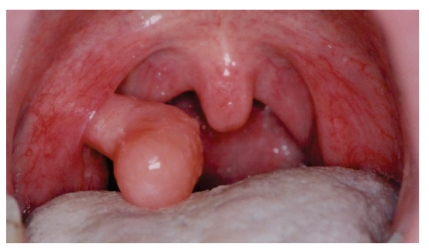
Primary lymphangioma of the tonsil: oral view of the tonsillar lymphangioma of the right tonsil of our patient.

On examination of his oral cavity, depression of the tongue revealed a large oval, pale pediculate mass protruding from the upper pole of the right tonsil and partially obstructing the airway (Figure 1). The rest of the oral cavity, nasopharynx, and laryngopharynx were normal. No significant neck lymphadenopathy was observed. Examination of head, ears, and nose was unremarkable. 

The patient underwent a right tonsillectomy under general anesthesia. The tonsil and the pediculate mass were removed by dissection, and hemostasis was performed by diathermy and ligation. The specimen was sent for histology. There were no postoperative complications, and the patient was discharged the following day. 

Histological examination showed macroscopically a tonsil 28 × 22 × 8 mm in size with an exophytic polypoid nodule measuring 26 × 10 × 8 mm. The cut surface of the tonsil showed normal crypt architecture. Microscopy examination of the polyp showed a core of loose fibrous tissue with dilated vascular spaces containing proteinaceous material and lined by sparse bland endothelial cells. There were foci of well-organized lymphoid tissue. The pedicle showed some well-formed blood vessels and fat. The surface was covered with squamous epithelium with varying degrees of degenerative activity. These features were consistent with lymphangioma of the tonsil. Abnormal lymphatics did not involve the deep tissues of the tonsil. Excision was complete. The adjacent tonsil showed no significant abnormality.

## 3. Discussion

Benign vascular tumors of the tonsils are rare. Lymphangioma of the tonsil is a rarity of which only few-well documented cases have been reported in the world literature [3–8]. Al Samarrae et al. [7] reported 2 cases of this disease in 1985, and in a review of the literature, they found only 6 well-documented cases previously reported. Since then, a few more cases in adults [8, 9], and occasionally in children, were published [10, 11]. Recently, Chen et al. [11] reported bilateral lymphangiomatous polyps of the palatine tonsils in a 4-year-old girl. However, most reports focused mainly on the pathologic and less on the clinical aspects of the tumor. The clinical behavior of the tumor is largely unknown, as most of these lesions are diagnosed histologically after surgical excision of the tonsils. In our patient, the tumor was large and pediculate, approaching the size of the tonsil, and although protruding into the oral cavity, the patient reported recent manifestation of symptoms, such as dysphagia and foreign body sensation. 

This tumor is classified as congenital vascular hamartomatous malformation rather than as a neoplasm. Lymphangiomas occur mostly during the first two decades of life and can sometimes be large. In the review of the literature, including our case, the mean age of the patient was 21.1 years old with a male to female prevalence 6 : 2.

Pathogenesis of tonsillar lymphangioma is uncertain, and three theories have been proposed to explain it [12].

**Failure of the Primordial Lymphatic Sacs to Drain into the Veins**

According to this theory, the failure of the lymphatic sacs to drain into the veins gives a dilation of the isolated lymphatic channels [13].

**Abnormal Sequestration of Lymphatic Tissue**

It has been hypothesized that abnormal sequestration of lymphatic tissue occurs early in embryogenesis. This theory helps explain the morphology of the more peripheral lesions, such as capillary and cavernous lymphangiomas [14].

**Abnormal Budding of the Lymphatics**

According to this theory, these aberrant buds lose their connections with the lymphatic primordial and eventually canalize to form lymph-filled cysts. These cysts maintain their ability to branch and grow, and do so in an uncontrolled, disorderly manner [15].

The most common presenting symptoms [1, 2] are dysphagia and sore throat. However, the presence of these tumors can be asymptomatic and their detection may be accidental. Most of the time, the tumor appears as a painless mass. If it is very large, as in our case, it can affect the surrounding vital structures to produce rhinolalia clausa, respiratory difficulty, stridor, difficulty in swallowing, excessive saliva in the oral cavity, or nausea.

The history and the clinical examination are important, but histological examination is needed to establish the diagnosis. Differential diagnosis should include unilateral tonsillar hypertrophy owed to malignant neoplasms, benign lesions, acute or chronic inflammations, or parapharyngeal masses which may give rise to apparent tonsillar enlargement due to medial displacement of the tonsil.

The treatment of tonsillar lymphangiomas is the complete surgical excision of the mass and the tonsil. There have been no reported instances of disease recurrence after complete excision.
